# Role of the Endogenous Antioxidant System in the Protection of *Schistosoma mansoni* Primary Sporocysts against Exogenous Oxidative Stress

**DOI:** 10.1371/journal.pntd.0000550

**Published:** 2009-11-17

**Authors:** Marina de Moraes Mourão, Nathalie Dinguirard, Glória R. Franco, Timothy P. Yoshino

**Affiliations:** 1 Departamento de Bioquímica e Imunologia, Instituto de Ciências Biológicas, Universidade Federal de Minas Gerais, Belo Horizonte, MG, Brasil; 2 Department of Pathobiological Sciences, University of Wisconsin, Madison, Wisconsin, United States of America; Biomedical Research Institute, United States of America

## Abstract

Antioxidants produced by the parasite *Schistosoma mansoni* are believed to be involved in the maintenance of cellular redox balance, thus contributing to larval survival in their intermediate snail host, *Biomphalaria glabrata*. Here, we focused on specific antioxidant enzymes, including glutathione-S-transferases 26 and 28 (GST26 and 28), glutathione peroxidase (GPx), peroxiredoxin 1 and 2 (Prx1 and 2) and Cu/Zn superoxide dismutase (SOD), known to be involved in cellular redox reactions, in an attempt to evaluate their endogenous antioxidant function in the early-developing primary sporocyst stage of *S. mansoni*. Previously we demonstrated a specific and consistent RNA interference (RNAi)-mediated knockdown of GST26 and 28, Prx1 and 2, and GPx transcripts, and an unexpected elevation of SOD transcripts in sporocysts treated with gene-specific double-stranded (ds)RNA. In the present followup study, *in vitro* transforming sporocysts were exposed to dsRNAs for GST26 and 28, combined Prx1/2, GPx, SOD or green-fluorescent protein (GFP, control) for 7 days in culture, followed by assessment of the effects of specific dsRNA treatments on protein levels using semi-quantitative Western blot analysis (GST26, Prx1/2 only), and larval susceptibility to exogenous oxidative stress in *in vitro* killing assays. Significant decreases (80% and 50%) in immunoreactive GST26 and Prx1/2, respectively, were observed in sporocysts treated with specific dsRNA, compared to control larvae treated with GFP dsRNA. Sporocysts cultured with dsRNAs for GST26, GST28, Prx1/2 and GPx, but not SOD dsRNA, were significantly increased in their susceptibility to H_2_O_2_ oxidative stress (60–80% mortalities at 48 hr) compared to GFP dsRNA controls (∼18% mortality). H_2_O_2_-mediated killing was abrogated by bovine catalase, further supporting a protective role for endogenous sporocyst antioxidants. Finally, *in vitro* killing of *S. mansoni* sporocysts by hemocytes of susceptible NMRI *B. glabrata* snails was increased in larvae treated with Prx1/2, GST26 and GST28 dsRNA, compared to those treated with GFP or SOD dsRNAs. Results of these experiments strongly support the hypothesis that endogenous expression and regulation of larval antioxidant enzymes serve a direct role in protection against external oxidative stress, including immune-mediated cytotoxic reactions. Moreover, these findings illustrate the efficacy of a RNAi-type approach in investigating gene function in larval schistosomes.

## Author Summary

Species of the human blood fluke *Schistosoma* are estimated to infect approximately 200 million people worldwide, resulting in loss of health, vitality and productivity mainly among the world's poorest inhabitants. Since snail intermediate hosts represent an essential part of the flukes' life cycle, an understanding of the strategies used by the intramolluscan schistosome larvae to survive within this host may provide novel approaches for disrupting larval development and thus transmission to humans. Anti-oxidant enzymes produced by the parasite *Schistosoma mansoni* are believed to play a critical role in the maintenance of cellular redox balance, contributing to larval survival in their snail host, *Biomphalaria glabrata*. In this study, we have incorporated a RNA interference approach attempting to knock down specific anti-oxidant enzymes, including gluthatione-S-transferases 26 and 28 (GST26 and 28), gluthatione peroxidase (GPx), peroxiredoxins 1 and 2 (Prx1/2) and superoxide dismutase (SOD), and to evaluate their endogenous anti-oxidant function in the sporocyst stage of *S. mansoni*. Results clearly demonstrated a significantly higher susceptibility of antioxidant double-stranded (ds)RNA-treated larvae to in vitro H_2_O_2_ treatment or hemocytic encapsulation compared to GFP dsRNA controls. Taken together, our findings support the hypothesis that endogenous expression and regulation of larval antioxidant enzymes serve a direct role in protection against external oxidative stress, including immune-mediated cytotoxic reactions.

## Introduction

Miracidial penetration and entry into the molluscan intermediate host represent a critical transition period in which the previously free-living larval stage is now confronted with a potentially hostile environment as it attempts to establish a viable infection [1],[2]. Miracidia of the human blood fluke *Schistosoma mansoni* shed their ciliated epidermal plates soon after entry into the host snail *Biomphalaria* spp., transforming to primary or mother sporocysts. It is during this time of transition and early sporocyst development that larvae are especially vulnerable to oxidative stress generated from products of oxidized plasma hemoglobin [3], or reactive oxygen or nitrogen species (ROS and RNS, respectively) resulting from hemocyte-mediated immune responses [4]–[7]. In such a potentially damaging environment, it is vital that parasites possess the capability of maintaining a redox equilibrium in order to counteract the effects of ROS/RNS generated both internally (products of endogenous metabolic oxidative reactions) and externally (environmental insults) [1],[8].

Recent studies have shown that *S. mansoni* larvae possess numerous enzymes involved in ROS metabolism and detoxification of oxidative products [9]–[14], and, like their adult stage counterparts [15]–[18], appear to complement each other to maintain the redox balance in the parasite. Included among these enzymes are the following: (i) glutathione-S-transferases 26 and 28 (GST26 and GST28) that function to neutralize potential membrane damage by the linked catalysis of glutathione (GSH) reduction with detoxification reactions involving thiol-conjugation to xenobiotics [19], (ii) peroxiredoxin (Prx1 and Prx2) that are involved in maintaining redox balance, by reducing hydrogen peroxide (H_2_O_2_) using a thioredoxin as an electron donor [20], (iii) superoxide dismutases (SOD), metalloenzymes responsible for catalyzing the dismutation of the superoxide radical to hydrogen peroxide as a defense mechanism against oxygen toxicity [21], and (iv) glutathione peroxidase (GPx), an H_2_O_2_-metabolizing enzyme that protects membranes from damage by phospholipid peroxidation [20],[22]. It is noteworthy that unlike most organisms, catalase, an enzyme responsible for H_2_O_2_ metabolism, is absent in *S. mansoni* [18],[23],[24], but is functionally replaced by Prx and GPx [16]. Interestingly, for schistosome GPx, whose H_2_O_2_-reactivity is typically very low in adult worms [8], exposure to the mammalian host environment induces enzyme activity and appears to be positively correlated to the parasite's resistance to oxidative stress [22]. In contrast to GPx, high levels of Prx activity are found in adult *S. mansoni* worms, and these enzymes are believed to be key components in maintaining redox balance, as well as are major contributors to antioxidant activity [16].

Previous findings have demonstrated that *in vitro* cultured *S. mansoni* sporocysts are highly sensitive to H_2_O_2_ toxicity [5], and that sublethal exposure of sporocysts *in vitro* to ROS, in particular H_2_O_2_, elicits an upregulation of genes encoding various antioxidant proteins [7],[11]. These data support the hypothesis that the primary sporocyst is capable of interfering with, or deactivating ROS-mediated damage, through activity of an endogenous antioxidant system [1]. However, to date, a functional role of specific antioxidant enzymes within intact larvae in providing protection against external ROS insults has not been demonstrated. Recently Mourão *et al.* [25] demonstrated consistent transcript knockdown for various antioxidant/redox-active detoxicant mRNA species in *S. mansoni* sporocysts using RNA interference as originally described [26]. These included transcripts for GST26 and 28, Prx1 and 2, and GPx. As a followup to these findings, the present study was conducted to determine the functional consequences of these induced antioxidant gene changes, especially their relevance to *S. mansoni* sporocyst interactions with the intermediate snail host *B. glabrata*.

## Materials and Methods

### Ethics statement

Research procedures involving mice used in the course of this study were reviewed and approved by the Institutional Animal Care and Use Committee (IACUC) at the University of Wisconsin-Madison under assurance no. A3368-01.

### *In vitro* cultivation of larval *Schistosoma mansoni*

The NMRI strain of *S. mansoni* was used for all experiments. *S. mansoni* eggs were isolated from livers obtained from mice harboring 7-week old infections, and miracidia hatched in an artificial “pond water” supplemented with antibiotics (50 µg/mL streptomycin and 60 µg/mL penicillin) [27]. Larvae were washed twice in ice-cold, sterile pond water by centrifugation, before being resuspended in Chernin's Balanced Saline Solution (CBSS; [28]), containing glucose and trehalose (1 g/L each) streptomycin and penicillin (50 µg/mL and 60 µg/mL, respectively). Miracidia were then counted and distributed into 48- or 96-well polystyrene tissue culture plates (Costar, Corning Incorporated, NY), at concentrations of **∼**500, 1000 or 8000 miracidia/well for oxidative stress experiments, immunocytochemistry or Western blot analyses, respectively. Finally, double-stranded RNAs were synthesized from isolated sporocyst cDNA using T7 RiboMAX Express RNAi Kit (Promega, Madison, WI), according to manufacturer protocol. Briefly, dsRNAs synthesis reactions were allowed to incubate for 16 hr at 37°C prior to DNAse treatment. DsRNA products were then extracted by phenol/chloroform and purified by precipitation with isopropanol. DsRNAs (50 nM final concentration) for specific antioxidant genes or green-fluorescent protein (GFP; specificity control dsRNA) were added to cultures containing 100 µL of CBSS for the oxidative stress assays and immunocytochemistry and 400 µL for the Western blot experiments. Because of the sequence and functional similarities of Prx1 and 2, dsRNAs for these transcripts were combined as a single treatment, designated hereafter as Prx1/2. Larvae were incubated for 7 days as previously detailed [25], after which time the functional consequences of dsRNA treatments were determined in functional assays described below. It should be noted that in a previous series of RNAi experiments conducted in parallel with the present study [25], a consistent, significant knockdown of steady-state transcript levels for each of the antioxidant genes currently under study was well documented. The only exception was the Cu/Zn superoxide dismutase (SOD) gene, in which larval exposure to SOD dsRNA resulted in a consistent increase, not knockdown, of SOD transcripts.

### Western blot analysis

To assess the effects of antioxidant dsRNA on the expression of specific proteins in sporocysts, we analyzed protein extracts of dsRNA-exposed sporocysts by Western immunoblot analysis [29] incorporating specific antibodies to two antioxidant species; namely SmGST26 (Cell Signaling Technology, Danvers, MA) and SmPrx1/2 (gift from Dr. D. Williams). Briefly, protein samples (∼8 µg) and Precision Plus Dual Color Marker (Bio-Rad, Bio-Rad Laboratories, Inc., Hercules, CA) were separated on 12.5% SDS-PAGE gels and transferred by semi-dry electroblotting (Amersham Biosciences) to nitrocellulose membranes (Bio-Rad). After blocking overnight in TBS (2.42 g Tris base, 8 g NaCl, pH 7.6) containing 5% bovine serum albumin (BSA), membranes were incubated in specific antibodies or a mouse anti-α tubulin antibody (serving as loading control, 1∶1000 dilution; Upstate Biotechnology Inc., Lake Placid, NY) for 16 hr at 4°C with gentle rocking. Membranes were then washed for 30 min in TBS-Tween (0.1%), and incubated for 1 hr in TBS-BSA (5%) containing either alkaline phosphatase (AP)-conjugated goat anti-rabbit IgG or AP-rabbit anti-mouse IgG at dilutions of 1∶10^4^ and 1∶5000, respectively (Promega, Madison, WI). The colorimetric immunoreactivity was detected with the chromogen 5-bromo-4-chloro-3-indolyl phosphate (BCIP) and nitro-blue tetrazolium (NBT) diluted in AP buffer (0.1 M Tris, 0.1 M NaCl, 0.05 M MgCl_2_, pH 9.5).

To quantify the observed immunoreactivity of each target protein in sporocysts treated with specific dsRNA and control GFP dsRNA, the intensities of reactive target bands were measured using Ultraviolet Transilluminator BioImaging Systems (UVP, Inc., Upland, CA) and normalized to the αtubulin band with LabWorks Image Acquisition and Analysis Software (version 4.6) in order to quantitatively evaluate the effects of antioxidant dsRNA treatment on specific protein levels. Three independent experimental replicates were performed and analyzed by Student's *t*-test, with significance set at *P*≤0.05.

### Immunocytochemistry

In order to compare *in situ* GST26 and Prx protein levels in antioxidant dsRNA-treated parasites, we prepared whole, intact sporocysts for immunofluorescent observations. All washing steps, in eppendorf tubes, were performed by centrifugation at 1600 rpm for 2 min and repeated 5 times, or as otherwise mentioned. Following transformation and *in vitro* cultivation (24 hr), sporocysts were washed 3 times in CBSS, to remove detached ciliated plates, prior transfer to siliconized-tubes containing 2% paraformaldehyde and 1% Triton-X100/sPBS. Larvae were fixed overnight at 4°C under gentle agitation, then washed in snail phosphate-buffered saline (sPBS; [30]) and resuspended in blocking buffer (5% normal goat serum + 0.02% sodium azide in sPBS) for 16 hr at 4°C. Rabbit-anti-GST26 or mouse anti-Prx1/2 primary antibodies, diluted at 1∶2000, and 1∶200, respectively, were then added to the larvae in fresh blocking buffer for 16 hr at 4°C under gentle agitation. This was followed by 5 washes, 10 min each, in sPBS, and resuspension in blocking buffer containing 4 µg/mL AlexaFluor 488-conjugated anti-rabbit/mouse antibody, 7 units/mL phalloidin-Alexa 546 and 10 µg/mL Hoechst 33258 dye (Invitrogen). Tubes containing samples were incubated for 16 hr at 4°C under constant rotation, followed by washing in sPBS, resuspension in 40 µl of sPBS and mounting on coverslips. A Nikon Eclipse TE2000 (Nikon Instrument Inc., Melville, NY) inverted epifluorescence microscope equipped with a Bio-Rad Radiance 2100 MP Rainbow Confocal/Multiphoton Imaging System (W.M. Keck Laboratory for Biological Imaging, Instrumentation, UW-Medical School) was used for specimen imaging and evaluation.

### Oxidative stress experiments

#### Peroxide toxicity

A series of *in vitro* experiments were designed to test the potential functional consequences of antioxidant protein knockdown in sporocysts treated with a sublethal concentration of H_2_O_2_. In preliminary tests, to determine our working sublethal peroxide concentration we exposed 7-day cultured GFP dsRNA-treated sporocysts to increasing amounts of H_2_O_2_ (Sigma-Aldrich, St Louis, MO) in a 96-well plate containing 0 µM (buffer only control), 5 µM, 10 µM, 25 µM, 50 µM, 100 µM, and 5 mM H_2_O_2_ in CBSS. Propidium iodide (PI; Invitrogen, Carlsbad, CA), used as a vital dye (5 µg PI/mL CBSS), was added at the time of viability scoring and the percentage of dead sporocysts, determined by intense staining with PI (PI-positive or PI+) was calculated at 24- and 48-hr post-treatment according to the following formula:
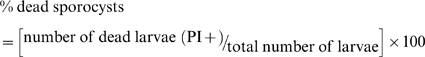



Larval mortality in ≤50 µM H_2_O_2_ was comparable to untreated sporocysts (CBSS alone) at both time intervals (data not shown), and therefore 50 µM was chosen as our sublethal H_2_O_2_ concentration.

#### Effect of antioxidant gene knockdown on sublethal H_2_0_2_-mediated sporocyst killing

To evaluate the functional relevance of the antioxidant enzymes GPx, GST26, GST28, Prx1/2 and SOD on parasite survival under oxidative stress conditions, 7-day dsRNA-treated sporocysts were exposed to 50 µM H_2_O_2_ for 4, 24 and 48 hr prior to evaluating larval viability. Freshly-hatched miracidia were axenically isolated and soaked in CBSS containing 50 nM of GPx, GST26, GST28, Prx1/2,SOD or control GFP dsRNAs and cultured for 7 days in 24-well plate as previously described [25]. Each treatment group was then divided into 3 wells of a 96-well plate: 2 of the wells were exposed to 50 µM of H_2_O_2_, and the third well was used as a no-treatment control (no H_2_O_2_). Cultures were incubated at 26°C and dead parasites (PI+) were counted at 4 h, 24 h and 48 h using an epifluorescent inverted microscope Nikon Eclipse TE 300 (Nikon Instruments Inc.). Data were represented as mean percentage dead sporocysts: 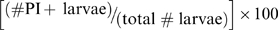, at each time point for each treatment group. Two-way ANOVA with Bonferroni post-test was used to compare the % mortality of antioxidant dsRNA-treated groups over exposure time interval to the GFP dsRNA-treated control group. Significance was set at *P*≤0.05; N = 5.

#### Catalase neutralization of H_2_O_2_-mediated oxidative stress

To verify that H_2_O_2_ is the major oxidizing agent responsible for initiating sporocyst death, dsRNA-treated larvae were prepared as described above and exposed to 50 µM H_2_O_2_ containing 0.1%, 0.05%, 0.025%, 0.0125% of bovine catalase (Sigma-Aldrich). Additional control cultures containing H_2_O_2_ alone (positive killing control) and catalase alone (catalase control) were concurrently run. All parasites were incubated at 26°C, and larval death assessed at 0, 4, and 48 hr post-treatment, using propidium iodide (Invitrogen). For each dsRNA treatment, the mean percentage mortality between H_2_O_2_-exposed, H_2_O_2_ + catalase-exposed and unexposed parasites with time was compared using Two-way ANOVA with Bonferroni post-test and with significance set at *P*<0.05 (N = 6).

#### Protective effect of antioxidants against in vitro hemocyte-mediated killing

To investigate the larval protective role of endogenous antioxidant molecules, sporocysts were treated with GPx-, Prx1/2-, GST26-, GST28-, SOD- and GFP-(control) dsRNAs for 7 days, followed by co-incubation with hemocytes of the susceptible NMRI strain of *Biomphalaria glabrata* in an *in vitro* cell-mediated cytotoxicity assay [5]. Because we wished to test for an effect of antioxidant knockdown on the efficacy of hemocyte-mediated killing, cells of the susceptible NMRI strain were used to determine if their basal level of killing efficiency could be significantly altered due to antioxidant knockdown. Eighteen snails (14–18 mm shell diameter) were used for each assay, in which the shell of each animal was dried, swab with 70% ethanol, and soaked in filter-sterilized “pond” water containing 60 µg/mL penicillin G, 50 µg/mL streptomycin and 25 µg/mL amphotericin B for 30 min. Snail shells were again dried and swabbed with 70% ethanol before headfoot bleeding as described in Sminia and Barendsen [31]. Whole hemolymph was pooled in a Petri dish on ice to facilitate removal of any extraneous shell debris or mucus, and then transferred to sterile 15-mL centrifuge tubes containing an equal volume of ice-cold CBSS.

The cytotoxicity assay described by Hahn *et al*. [5] was used with some modifications. Approximately 500 µL of hemolymph was gently aliquoted in siliconized-eppendorf tubes containing an agarose plug (0.2% agarose) and 50 µL of 5% Ficoll (Sigma-Aldrich Inc.) in incomplete or I-Bge (24% Schneider's *Drosophila* medium, Invitrogen; 0.5% lactalbumin hydrolysate, Sigma-Aldrich; 7.2 mM galactose). Tubes were centrifuged at 20×*g* for 17 min to isolate and concentrate hemocytes. Cell-free plasma and Ficoll were removed and discarded, followed by resuspension of hemocytes in sterile CBSS and redistribution in equal aliquots to wells of a 16 CultureWell™ Chambered Coverglass slide (Invitrogen) containing approximately 100 dsRNA-treated sporocysts in I-Bge medium. After 1 hr of co-cultivation at 26°C, 3 µL of propidium iodide (PI; 5 µg/mL) (Invitrogen) were added to a subset of wells and the total number of sporocysts (Nomarski DIC optics) and number of dead sporocysts, those exhibiting positive PI staining (PI+; epifluorescence microscopy), per treatment were counted in order to establish an initial mortality rate. Enumeration of total and dead sporocysts was again determined at 24 hr post-cultivation. The percentage of sporocysts killed after 24 hr of co-culture was calculated for groups of larvae treated with antioxidant dsRNAs and control GFP dsRNA, and compared according to the following formula [5]:
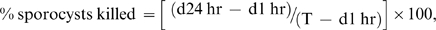
where “d” = # dead sporocysts at the indicated time interval (1 or 24 hr) and “T” = total # sporocysts. Statistical analyses were performed using Student's *t*-test in which the % sporocyst death at 24 hr was compared between antioxidant dsRNA-treated and control GFP dsRNA-treated groups. Significance was set at *P*≤0.05 (N = 4)

## Results

Previous work in our lab has established a consistent and specific pattern of altered antioxidant transcript expression in primary sporocysts after 7 days of double-stranded (ds) RNA exposure [25]. Specifically, statistically significant knockdown of *S. mansoni* GST26, GST28, GPx, and Prx1/Prx2 transcript levels, and an unexpected robust increase in those of SOD were observed in dsRNA-treated larval populations. To further explore the functional relevance of these enzymes in this parasite model, we conducted experiments to determine how antioxidant dsRNA exposure affected gene expression at the protein level (for selected enzymes), and whether a functional association could be established between antioxidant gene knockdown and parasite survival in presence of stressors such as reactive oxygen species (H_2_O_2_) or encapsulating hemocytes.

**Figure 1:**
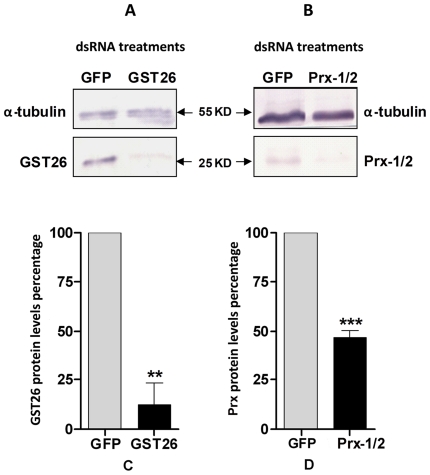
Western blot analyses of SDS-PAGE separated extracts of *Schistosoma mansoni* sporocysts. Larvae were cultured for 7 days in CBSS containing GFP, GST26, or Prx1/2 dsRNA, followed by probing with specific anti-GST26 (Fig. 1A), anti-Prx1 (Fig. 1B) or sample loading control anti-α tubulin antibodies. Using anti-α tubulin reactivity to normalize sample loads, scanning densitometry was used to quantify immunoreactive GST26 (Fig. 1C) and Prx1 (Fig. 1D) intensities in specific dsRNA-treated vs. GFP dsRNA control sporocyst groups. Both GST26 and Prx protein levels were significantly knocked down by 80% and 50%, respectively, when compared to the GFP dsRNA treatment. ** *P*<0.01; *** *P*<0.001; N = 3.

To verify that specific dsRNA treatments had a predicted downregulating effect on sporocysts at the protein levels, Western blot analyses were performed on sporocysts treated with dsRNA for GST26, Prx1/2 and GFP (control) using antibodies specifically against *S. mansoni* GST26 and Prx1/2 [20]. In all experiments a crossreactive anti-α tubulin antibody served as a loading control. As shown in Figure 1, proteins extracted from GFP dsRNA-treated sporocysts (specificity control) presented two distinctive bands at ∼26 and 55 kDa, corresponding to GST26 and α tubulin, respectively. However, although larvae treated with GST26 dsRNA also exhibited the 55 kDa α tubulin protein, little immunoreactivity was observed at 26 kDa, suggesting an RNAi-induced GST26 protein knockdown (Fig. 1A). Quantification of band intensities by scanning densitometry, using anti-α tubulin reactivity to normalize protein loads in both treatment samples, confirmed that GST26 protein levels were significantly reduced (by ∼80%) in GST26 dsRNA-treated sporocysts compared to the nonspecific GFP dsRNA control group (Fig. 1C). Similarly, although not as dramatic, larval exposure to Prx1/2 dsRNA also exhibited a significant ∼50% decrease in protein level compared to the GFP control treatment by semi-quantitative Western blot analysis (Figs. 1B and D).

**Figure 2:**
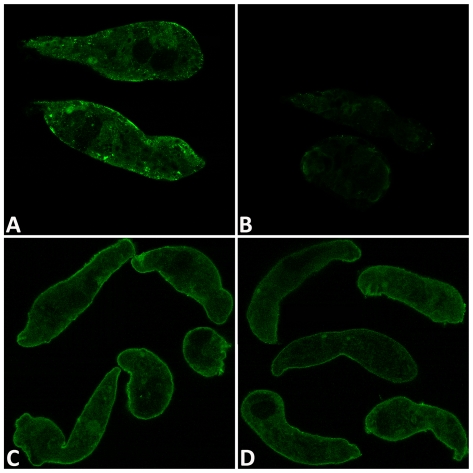
Representative confocal epifluorescent photomicrographs of *Schistosoma mansoni* sporocysts showing immunolocalization of anti-GST26 and anti-Prx1 antibodies after cultivation in medium containing GST26, Prx or control GFP dsRNA. Fluorescence specific to anti-GST26 reactivity (green) observed in GST26 dsRNA-treated larvae (Fig. 2B) was noticeably reduced compared to the nonspecific GFP dsRNA control sporocysts (Fig. 2A), consistent with the high protein knockdown (∼80%) seen in Western blot analysis. By contrast, little difference in fluorescence levels was observed between the nonspecific GFP dsRNA control-treated (Fig. 2C) and Prx1/2 dsRNA-treated (Fig. 2D) sporocysts, reflecting the relatively small decrease (∼50%) observed in immunoblot protein levels. N = 3.

Consistent with Western blot analyses, *in situ* confocal observations of anti-GST26 localization in GST26 dsRNA-exposed and control GFP dsRNA-treated sporocysts revealed contrasting expressions of immunoreactivities. Anti-GST26 antibodies strongly reacted with endogeneous *S. mansoni* GST26 in sporocyst controls (Fig. 2A), but was much reduced in those treated with GST26 dsRNA (Fig. 2B), indicating a RNAi-mediated GST26 protein knockdown. Immunolocalization of anti-Prx1/2, however, revealed little difference in observed staining intensities between the GFP and Prx dsRNA-treated groups (Figs. 2C and 2D, respectively), except for a slight decrease in surface immunoreactivity in Prx-treated sporocysts. This also is consistent with the smaller knockdown effect of dsRNA exposure on Prx protein expression seen in immunoblot analysis (Fig. 1B).

**Figure 3:**
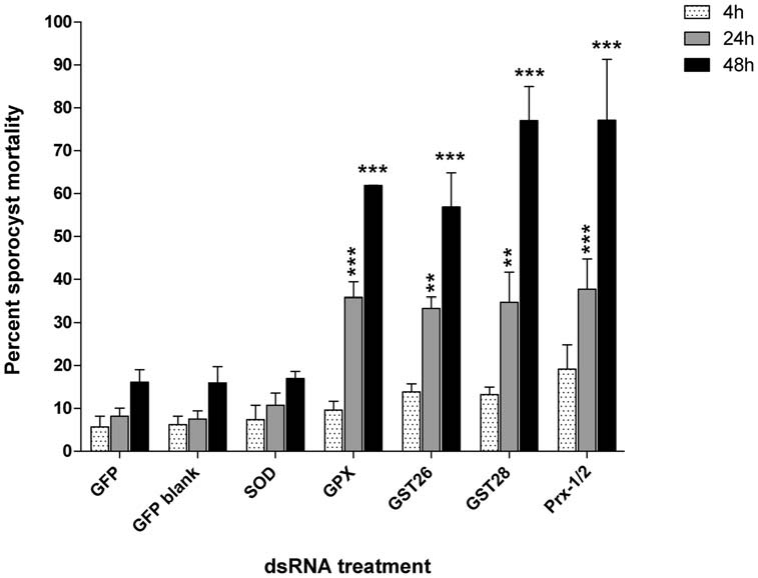
Graphic representation of the effect of exogenous H_2_O_2_ exposure on *Schistosoma mansoni* sporocysts following treatment with dsRNAs for GFP (specificity control), SOD, GPx, GST26, GST28 and Prx1/2. Double-stranded RNA-reated sporocysts were exposed to 50 µM H_2_O_2_ for 4, 24 and 48 hrs (stippled, grey and black bars, respectively). Knockdown of larval GPx, GST26, GST28 and Prx1/2 antioxidants increased sporocyst mortality after 24 and 48 hr under oxidative stress conditions when compared to GFP dsRNA-treated or no treatment controls. Note that sporocysts treated with SOD dsRNA showed no difference in susceptibility to H_2_O_2_ oxidation at any of the time points compared to controls. **P<0.001; ***P<0.0001; N = 4.

In order to evaluate the effects of a potential loss of antioxidant activity in sporocysts due to dsRNA-induced antioxidant knockdown, we exposed groups of treated parasites to a range of hydrogen peroxide (H_2_O_2_) concentrations. In these preliminary tests 50 µM H_2_O_2_ was determined to represent a sublethal dosage under our experimental conditions (% larval death was not significantly different from control groups), whereas mortality rates significantly increased at 100 µM and higher H_2_O_2_ concentrations (data not shown). As shown in Figure 3, none of the dsRNA-treated sporocysts exhibited significant increases in H_2_O_2_-mediated mortality when compared to the GFP control treatments after 4 hr of exposure. However, at 24 and 48 hr sporocysts in all dsRNA-treatments, except the SOD dsRNA-exposed group, displayed significant increases in mortality with an average of 35% sporocyst death compared to 8% in control treatments after 24 hr, and 60 to 80% mortalities, compared to ∼18% in control treatments, at 48 hr post treatments (F_dsRNA_ = 28.21, *P*≤0.0001; F_Time_ = 84.71, *P*≤0.0001, N = 4). In contrast to other antioxidant treatments, sporocysts exposed to SOD dsRNA exhibited a H_2_O_2_-mediated mortality rate similar to that of control treatments at all time points (Fig. 3). See Figure 3 legend for means comparisons using Bonferroni's post-test.

**Figure 4:**
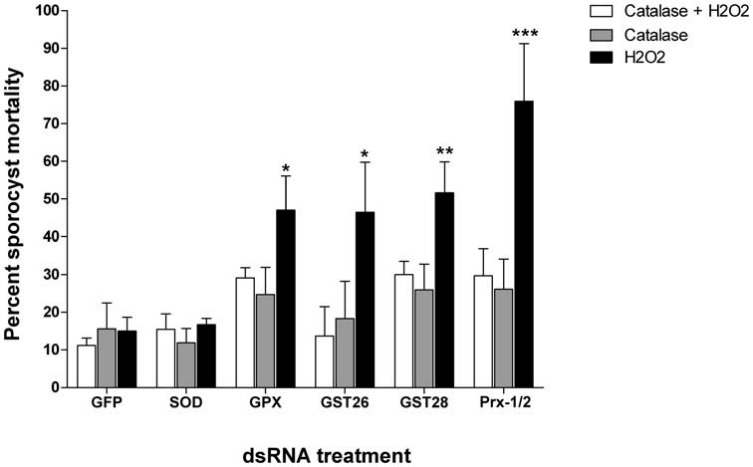
Effects of catalase on H_2_O_2_-mediate killing of GPx, GST26, GST28, SOD, Prx1/2 dsRNA-treated and control GFP dsRNA-treated *Schistosoma mansoni* sporocyst *in vitro*. After 7 days of dsRNA incubation sporocysts were exposed to H_2_O_2_ alone, catalase alone or catalase combined with H_2_O_2_ for 48 hr followed by evaluation of sporocyst death using propidium iodide staining. Significant increases in H_2_O_2_–mediated mortality was abrogated in the presence of bovine catalase [H_2_O_2_+catalase] showing that H_2_O_2_ was the primary source of larval killing in antioxidant dsRNA-treated sporocysts, with the exception of SOD, *P<0.05; **P<0.001; ***P<0.0001; N = 6.

To confirm that sporocyst death was specifically due to H_2_O_2_ as an exogeneous oxidative stressor, we exposed dsRNA-treated sporocysts to 50 µM H_2_O_2_ in presence or absence of bovine catalase or to catalase only (no H_2_O_2_ control), and evaluated sporocysts mortality in all treatments after 48 hr. Overall ANOVA indicated a significant effect of dsRNA treatment and H_2_O_2_-exposure (F_dsRNA_ = 7.44, *P*≤0.001; F_Oxid_ = 15.33, *P*≤0.0001, N = 6). Within each treatment group, the percent mortalities for sporocysts exposed to GPx, GST26, GST28 and Prx1/2 dsRNAs were very similar when incubated in H_2_O_2_+catalase or catalase only (*t* values ranging from 0.23–1.74; all nonsignificant) (Fig. 4). These results are in contrast to the effects of exposure to H_2_O_2_ alone (positive killing control), in which mortality rates for sporocysts treated with the same antioxidant dsRNAs were significantly higher (ranging from 50–75%) when compared to 25% average sporocyst death in the catalase treatment groups (see Fig. 4 for Bonferroni's post-test comparisons). As previously observed, SOD dsRNA-treated larvae, again showed no difference in mortality rates between the different treatments, nor when compared to the control GFP dsRNA group.

**Figure 5:**
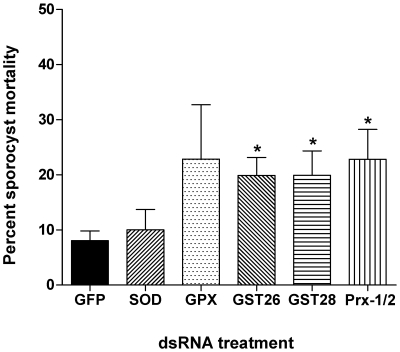
*In vitro* cell-mediated cytotoxicity (CMC) assay results. *Schistosoma mansoni* sporocysts, cultured for 7 days in medium containing antioxidant (GPx, Prx1/2, GST26 and 28, SOD) or control GFP dsRNA were incubated for 24 hr with plasma-free hemocytes from the susceptible NMRI snail strain of *Biomphalaria glabrata* followed by assessment of larval mortality by propidium iodide staining. Co-culture of GST26, GST28 and Prx1/2 dsRNA-treated sporocysts with snail hemocytes resulted in small, but significant increases in percent larval mortality when compared to GFP dsRNA controls. The GPx dsRNA-treatment exhibited a nonsignificant increase in larval killing, while mortality of SOD dsRNA-treated sporocysts showed no difference compared to GFP dsRNA controls. *P≤0.04; N = 4.

Finally, in order to evaluate the effect of dsRNA antioxidant knockdown on snail hemocyte-sporocyst interactions *in vitro*, dsRNA-treated sporocysts were co-cultured with isolated hemocytes from the susceptible NMRI strain of *Biomphalaria glabrata*. After 24 hr of sporocyst-hemocytes incubation in an *in vitro* cell-mediated cytotoxicity assay [5], we observed that dsRNA knockdown of GST26 (*t* = 2.50, *P*≤0.01), GST28 (*P*≤0.0461) and Prx1/2 (*t* = 3.17, *P*≤0.04) resulted in small, but statistically significant increases in larval death, averaging ∼20% compared to ∼8% mortality in the GFP dsRNA control group (Fig. 5). Note that sporocysts treated with GPx dsRNA also showed an increase in mean mortality rate, but was not statistically significant when compared to the GFP control parasites. As observed in previous experiments, sporocysts treated with SOD dsRNA exhibited no difference in mortality compared to the GFP-treated control sample.

## Discussion

Enzymes involved in cellular redox pathways, which include proteins with antioxidant activities, are believed to be essential components regulating *B. glabrata*/*S. mansoni* molecular interaction [1],[2]. It is now well recognized that certain strains of *B. glabrata* snail immune cells or hemocytes produce substantial amounts of reactive oxygen [4],[5] and nitrogen [6] species as a consequence of stimulation by known activators of ROS/RNS or when encountering *S. mansoni* sporocysts, and that sporocysts are exquisitely sensitive to ROS-mediated killing, especially to H_2_O_2_. Moreover, in a series of followup studies, Bayne and co-workers have implicated a Cu/Zn-superoxide dismutase (SOD1) as a key enzyme involved in oxidative killing activity by hemocytes of resistant (R) strains of *B. glabrata* snails. Their studies demonstrated that (1) SOD transcript expression and enzyme activity are higher in certain R vs. susceptible (S) snail hemocytes [32] and this correlates with greater H_2_O_2_ production in the R strain [33], (2) *B. glabrata* SOD1 is comprised of 3 alleles, of which one (B allele) is significantly associated with R snails [34], and (3) SOD1 B allelelic expression is higher in R hemocytes than those of the S strain [35]. Based on their findings it is suggested that snail strain differences in SOD hemocyte expression may be causally linked to the observed S and R strain phenotypes. Because SOD catalyzes the conversion of superoxide to cytotoxic H_2_O_2_ it is reasoned that upregulation of the SOD1 gene and its resultant heightening of SOD enzymatic activity in R hemocytes may represent a possible mechanism for the differential larval killing response by R vs. S snail hemocytes [2].

While snail hemocytes produce H_2_O_2_ as an anti-parasite effector molecule, evidence also strongly supports the presence of an active antioxidant system in early developing *S. mansoni* sporocysts [11]–[13]. Catalase gene homologues were not found in recent searches of the *S. mansoni* genomic and EST databases, and this is consistent with earlier findings [16],[23],[24] indicating that these parasites must possess alternative means for neutralizing H_2_O_2_ and other ROS. As clearly demonstrated in mammalian stages of *S. mansoni*, this is accomplished by a thiol-dependent redox system involving thioredoxin glutathione reductase (TGR) as the central enzyme driving redox reactions [36]. Similarly, early intramolluscan larval stages also express redox genes, including TGR, thioredoxin, Cu/Zn SOD, GPx, Prx and GST [7], [11]–[13],[37], and in the case of GPx [7] and Prx1 and 2 [11], sporocyst expression levels are dramatically increased in response to ROS exposure. In addition, Cu/Zn SOD, GST26 and 28 and Prx were recently identified in larval transformation proteins (LTP) released during *in vitro* transformation of miracidia to sporocysts, demonstrating not only the synthesis of these antioxidants by miracidia, but also their active release during larval infection [9],[14]. Implied in these findings is the notion that antioxidant LTPs may be playing a potential protective role during early parasite development. This prospect of larval-protective antioxidants was given further credence by Vermeire and Yoshino [11] who demonstrated that Prx1/2 in LTP can function as scavengers of exogenous H_2_O_2_ suggesting the potential importance of excreted antioxidants as a sporocyst defense mechanisms.

In this study, we provide the first evidence for a functional role of the endogenous antioxidants GPx, Prx and GSTs in the survival of *S. mansoni* sporocysts confronted with exogenous oxidative stress. By successfully knocking down antioxidant transcript/protein levels using an RNAi-type approach, we were able to characterize the impact of introduced molecular H_2_O_2_ and presumed ROS produced during hemocyte encapsulation reactions on survival of intact primary sporocysts of *S. mansoni*. In a previous companion study that was run in parallel with the current experiments [25] we showed that larval treatment with double-stranded RNA (dsRNA) for all of the antioxidants, except SOD, produced a consistent, significant and specific transcript knockdown in sporocysts. In the present study, consistent with the transcript knockdown seen earlier, we demonstrated a dsRNA-associated decrease in GST 26 and Prx1/2 protein levels using specific antibodies in a semi-quantitative Western blot assay. This protein knockdown effect was supported by immunocytochemistry (ICC) in the case of GST26, but not as clearly for Prx. Importantly, the dsRNA-mediated decrease in GST26 and Prx protein content correlated well with significant increases in sporocyst mortality at 24 and 48 hr post-H_2_O_2_ exposure compared to the dsRNA control groups, implying a functional role for endogenous GST26 and Prx in the protection of primary sporocysts against external oxidative stress. Although lack of specific antibodies to the other antioxidants precluded a complete analysis of the other RNAi targeted genes used in this study, we continued to see a consistent correlation between dsRNA-induced decrease in transcript levels [25] and sporocyst survival patterns for larvae treated with GST28 and GPx dsRNA that were similar to those treated with GST26 and Prx1/2 dsRNAs. Indeed, compared to the untreated and GFP dsRNA controls, exposure of antioxidant dsRNA-treated sporocysts to a sublethal concentration of H_2_O_2_ *in vitro* resulted in dramatic decreases in parasite survival in all treatment groups except SOD, supporting the notion that GST28 and GPx, similar to Prx and GST26, also are capable of enhancing sporocyst survival in an oxidative environment.

These new findings are consistent with the extensive and ongoing work on the redox mechanism in the adult stage of *S. mansoni*, in which an active thiol-dependent redox maintenance system revolves around a thioredoxin glutathione reductase (TGR; [36]), a single enzyme that combines the activities of two enzymes, thioredoxin reductase and glutathione reductase, present in mammals [17]. Schistosome TGR is responsible for maintaining the reduced and active states of both thioredoxin (TR) and glutathione (GSH), allowing them to activate several Prxs and GPx, which in turn are capable of reducing H_2_O_2_ and other hydroperoxides [8]. Furthermore, in a more recent study, Sayed and coworkers [16] showed that Prx activity was essential to *S. mansoni* adult worm survival *in vitro*, further supporting the importance of maintaining a steady supply of this, and other antioxidant enzymes by *S. mansoni* adults. It appears that, like adult worms, early intramolluscan stages also must rely on robust endogenous system of antioxidant production that allows the parasite to overcome oxidative stress from both internal and external sources.

In addition to the antioxidant protective role of *S. mansoni* sporocysts in the presence of exogeneously introduced oxidative stress, we observed a similar survival pattern in dsRNA antioxidant-treated sporocysts that have come in contact with hemocytes from the susceptible NMRI *B. glabrata* strain. Our rationale for incorporating susceptible snail hemocytes in these experiments was to test the hypothesis that reducing the antioxidant capacity of sporocysts would increase their vulnerability to sublethal levels of ROS normally produced by NMRI snail hemocytes in *in vitro* cell-mediated cytotoxicity (CMC) assays [5],[38]. In this *in vitro* biologically-relevant context, we demonstrated a significant protective role of Prx and GSTs in sporocysts during hemocyte interactions. Co-culture of plasma-free hemocytes from susceptible NMRI snails with Prx, GST26, and GST28 dsRNA-treated sporocysts induced an increase in sporocyst mortality (to ∼20%) within 24 h of initial contact, when compared to GFP dsRNA-treated control group (8%). GPx dsRNA-treated sporocysts also showed a comparable increase in hemocyte-mediated killing, but high variance in replicate values rendered the increase nonsignificant. Thus the protective role of GPx against hemocyte-mediated ROS attack still remains to be proven. Taken together, however, our overall results suggest that ROS production in susceptible snail hemocytes is capable of overpowering antioxidant-deficient parasites. Zelck and Janowsky [7] hypothesized that susceptible snails generate relatively small amount of ROS, which in turn may induce antioxidant production in schistosomes, effectively neutralizing snail-generated ROS. In this study, we have demonstrated that effectively reducing their antioxidant enzyme capacity, sporocyst survival, when confronted by a usually benign hemocyte challenge, is significantly reduced, thus supporting the critical importance of the endogenous antioxidant system in establishing viable larval infections within the susceptible snail host.

Finally, a major exception to our present finding of enhanced larval susceptibility to oxidative stress by redox proteins was signal peptide (SP) Cu/Zn SOD [39]. In this case Cu/Zn SOD dsRNA treatment consistently had no effect on parasite survival whether in the presence of sublethal H_2_O_2_ or encapsulating hemocytes. These differing effects of SOD dsRNA exposure may have been predicted as treated *S. mansoni* sporocysts consistently displayed extreme elevations, rather than knockdown in transcript levels [25], indicating a strong induction of SOD gene expression in these larval stages. At present, the signaling mechanisms involved in this response are not known although, as suggested by Zelck and Von Janowsky [7] and Vermeire and Yoshino [11], sporocysts may be sensing ROS levels (including H_2_O_2_) and responding by upregulating protective antioxidant proteins. It is speculated that larval treatement with SOD dsRNA may have caused an initial downregulation of SOD transcripts that then led to a compensatory triggering of SOD over-expression. However, as shown in other systems, small interfering dsRNA also can trigger activation of transcription [40] and, therefore, could also represent a likely mechanism [25]. Its unusual expression pattern not withstanding, results indicate that hyperexpression of the SOD gene in *S. mansoni* sporocysts appeared to have a “neutral” effect on dsRNA-treated larvae (i.e., an effect similar to control dsRNA treatment) (present study). This does not necessarily imply that SOD has no role to play in maintaining redox balance within sporocysts both internally or in response to exogenous ROS sources. However, the mechanisms by which this is accomplished are currently unknown and represent the subject of further followup investigations in our lab.
